# Antibacterial effect of silver diamine fluoride and potassium iodide against *E. faecalis*, *A. naeslundii* and *P. micra*

**DOI:** 10.1186/s12903-021-01531-1

**Published:** 2021-04-07

**Authors:** Benjamín Briseño-Marroquín, Yasmine Ismael, Angelika Callaway, Christian Tennert, Thomas Gerhard Wolf

**Affiliations:** 1 Department of Restorative, Preventive and Pediatric Dentistry, School of Dental Medicine, University of Bern, Freiburgstrasse 7, 3010 Bern, Switzerland; 2 Department of Periodontology and Operative Dentistry, University Medical Center of the Johannes Gutenberg-University, Mainz, Germany

**Keywords:** Bactericidal effect, *A. naeslundii*, *E. faecalis*, *P. micra*, Potassium iodide, Silver diamine fluoride

## Abstract

**Background:**

The aim of this study was to determine in vitro the bactericidal potential of 38% silver diamine fluoride (SDF) alone, potassium iodide (PI) alone, and the two in combination (SDF + PI) against three bacterial species commonly found in root canal samples (*Enterococcus faecalis*, *Actinomyces naeslundii* and *Parvimonas micra*).

**Methods:**

The potential bactericidal rates for SDF, PI and SDF + PI against *E. faecalis*, *A. naeslundii* and *P. micra* were calculated as reduction of bacteria colony forming units.

**Results:**

The bactericidal potential of SDF was at 99.97–100% against *E. faecalis* and 100% against *A. naeslundii* and *P. micra*. SDF + PI showed a 100% bactericidal effect against *P. micra*, 99.89–99.98% against *E. faecalis* and 99.98–100% against *A. naeslundii*. The bactericidal effect of PI was 99.51–99.98% against *E. faecalis*, 99.27–99.95% against *A. naeslundii* and 99.93–100% against *P. micra*. The differences between controls and bacteria exposed to the antibacterial agents were statistically significant (*p* < 0.05).

**Conclusions:**

SDF had an effective bactericidal effect against the examined bacteria. However, the limitations of this in vitro study do not allow a recommendation of the employment of these solutions as root canal irrigants. Additional investigations are necessary to assess their endodontic clinical applicability.

## Background

Various types of injuries are the cause of pulp inflammation, which can ultimately lead to irreversible pulpitis and consequently through contamination to pulp necrosis [1]. Entry paths for microorganisms into the tooth pulp system are mainly caries, periodontal disease [1] or trauma [2]. Dahlén [3] reported that bacteria such as *lactobacilli, streptococci, actinomyces* and Gram-negative obligate anaerobes are often the cause of this type of inflammation.

The removal of inflamed and/or necrotic pulp tissue, and thus, of established bacteria is the main purpose of the mechanical and chemical shaping and cleaning process of the root canal system. Mechanical preparation of the root canal ensures shaping of the root canal; yet, a complete removal of infected and/or non-infected material cannot be accomplished. For almost a century the chemical cleaning of the root canal system has been recommended [4]. The standard solution for this purpose is sodium hypochlorite, which is recommended in concentrations from 0.5 to 5.25% [5], being the only root canal bactericidal irrigating solution capable of dissolving tissue [6, 7]; it also possesses also a bleaching effect [8] and it can be used in combination with EDTA, which is a chelator capable of removing smear layer from the root canal walls [9]. Another commonly used irrigating solution in endodontic treatment is chlorhexidine which also has a bactericidal effect [10]. It has been shown that chlorhexidine has a bactericidal effect against *Enterococcus faecalis*, [11] which is frequently found during endodontic re-treatment.

As early as 1917, Howe [12] reported that silver in form of an ammoniacal silver nitrate solution had a sterilizing effect on infected coronal as well as root dentin. Different research groups have investigated the bactericidal effect of silver diamine fluoride in dentistry and reported that it has a bactericidal effect against bacteria found in root canal infections such as *Streptococcus mutans*, *Actinomyces naeslundii* and *Enterococcus faecalis* [13–15]. The silver-diamine based product Riva Star is a two-component system: silver diamine fluoride and potassium iodide. However, it has been, so far, clinically employed as a desensitizer of sensitive dentin [16]. Thus, the aim of this study was to investigate in vitro the bactericidal properties of silver diamine fluoride and potassium iodide, alone and in combination in different concentrations against three bacteria commonly found in root canal samples: two facultatively anaerobic strains, coccal (*E. faecalis*) or rod-shaped (*A. naeslundii*) and an obligately anaerobic one (*P. micra*). *Actinomyces naeslundii* can be found in carious dentin and in root caries [17, 18] thus also in root canal systems [19]. *Parvimonas micra* is more frequently isolated from periodontal pockets [20]; yet, it can also be found in root canal systems [21]. *Enterococcus faecalis* occurs in gastrointestinal tracts of humans and other mammals [22]. According to Gold et al. [23] it gains access to the oral cavity through food ingestion and is often isolated from infected endodontically treated teeth [11, 24].

## Methods

Riva Star (SDI Limited, Bayswater, Australia) is a two-phase (38% silver diamine fluoride [SDF] and potassium iodide [PI]) tooth desensitizing and cavity cleanser system. The potential bactericidal effect of SDF and PI, as well as a combination of the two solutions (SDF + PI) was tested using three bacterial strains belonging to species commonly found in endodontic infections. Two strains were obtained lyophilized from the German Collection of Microorganisms and Cell Cultures GmbH (DSMZ, Braunschweig, Germany), the facultatively anaerobic *Enterococcus faecalis* DSM 20376 and the obligately anaerobic *Parvimonas micra* DSM 20468. The third strain, also facultatively anaerobic, *Actinomyces naeslundii* A65 was obtained from the Centre for Hygiene and Medical Microbiology, Bonn, Germany. *E. faecalis* 20376 and *A. naeslundii* A65 were grown as liquid cultures in Schaedler bouillon (BBL Schaedler Broth, Becton Dickinson and Company, Sparks, MD, USA) or on Schaedler agar plates. *P. micra* 20468 was grown in Anaerobe Basal Broth (Oxoid Ltd., Basingstoke, Hampshire, UK) and on agar plates prepared from the broth. Anaerobic conditions were obtained through incubation of the bacterial cultures in an anaerobic jar containing a GasPak envelope producing H_2_ and CO_2_ (GasPak EZ; Becton Dickinson and Company, Sparks, MD, USA) for 24 h (*E. faecalis* 20376), 48 h (*A. naeslundii* A65) or 72 h (*P. micra* 20468), at 37 °C (Heratherm Incubator, Thermo Scientific, Langenselbold, Germany). An inoculum of 40 µl was used for the liquid cultures. Purity of the bacterial cultures was confirmed each time by taking a sample, placing it on a microscope slide and visually analyzing it using a phase contrast microscope (Carl Zeiss, Jena, Germany; 1250x).

A modified agar diffusion technique was used to obtain preliminary information about a potential antibacterial effect of the liquids from Riva Star. 100 µl of the bacterial cultures were applied to the respective agar plates with a spreader, using a turntable. Then sterile paper discs (Oxoid Ltd, Basingstoke, Hampshire, UK) were placed on top of the agar, and 10 µl of the liquids were applied to the paper discs. The antibacterial effect of SDF alone was tested undiluted, and to simulate possible residual fluids in the root canal system, diluted to 19%, to 9.5% and to 3.8% (100% SDF = 38%; 50% = 19%; 25% = 9.5%; 10% = 3.8%) with sterile saline; PI was tested only undiluted. For the combined effect, 5 µl of SDF (undiluted, diluted to 19%, to 9.5% and to 3.8%) and 5 µl of the undiluted PI were applied together to the sterile paper discs. After incubation for 24–72 h, depending on the bacterial strain as described above, the diameters of the inhibition zones were measured and expressed in centimeters.

In an effort to simulate clinical conditions that would allow applying the solutions directly to the bacteria with the micro brushes provided with the Riva Star system, an in vitro model was developed. Individual bacterial colonies of similar size were obtained by streaking an inoculation loop with the respective bacteria on the corresponding agar plates, using the quadrant streak technique. The plates were then incubated as described above, depending on the bacteria, for 24, 48 or 72 h at 37 °C. For each bacterial strain four colonies of the same size were needed for each experiment, one as untreated control, one in contact with SDF, one with PI and one with SDF + PI. To determine the viable count of the controls, a colony was picked and placed into a sterile Eppendorf tube containing 1 ml of sterile saline and mixed on a vortex (Janke & Kunkel IKA-Labortechnik, Staufen, Germany). Serial dilutions of 10^–1^ dilution steps were performed until dilutions of up to 10^–6^ were reached. 100 µl of each dilution were then plated on the respective agars and incubated as described above. The number of colony-forming units (CFUs) on plates containing 30–300 colonies was then counted and converted to the number of organisms per milliliter. The remaining three colonies were placed on sterile paper discs, and undiluted SDF, PI or SDF + PI was applied with the silver or green micro brushes provided with the Riva Star system. When the combined solutions were used, SDF was applied first and then PI. After 30 s of exposure time which is recommended by the manufacturer, each paper disc was placed into an Eppendorf tube with 1 ml sterile saline and processed as described above for the controls. The numbers of colony forming units (CFUs) was counted on plates containing 300 or fewer colonies and converted to the number of organisms per milliliter.

**Fig. 1:**
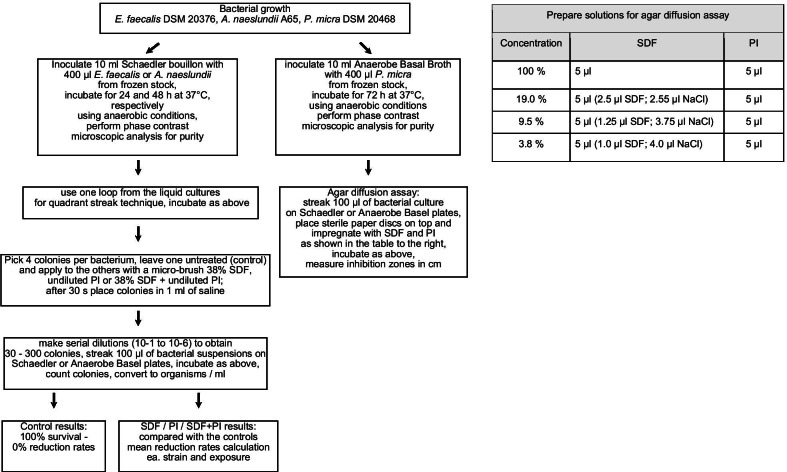
Outline of the study protocol. The figure depicts the flow of the laboratory research protocol through the study

The number of viable bacteria (CFUs) for the untreated controls was considered as 100% survival or 0% reduction rate. The numbers of CFUs of the treated bacteria were then compared with the controls, and reduction rates in percent were calculated for each strain and each exposure to SDF, PI and SDF + PI. The study design is depicted in Fig. 1.

### Statistical evaluation

A statistical evaluation of the sizes of the inhibition zones (mean, median, standard deviation, minima, maxima and percentile) was carried out (SPSS Version 22 for Windows; Chicago, IL, USA) in cooperation with the Institute for Medical Biometry, Epidemiology and Informatics (IMBEI, University Medical Center of the Johannes Gutenberg-University, Mainz, Germany). The effect of the undiluted solutions of the SDF, PI and SDF + PI groups were compared for each bacterial strain by means of the nonparametric test for independent samples using the Kruskal–Wallis test (*p* < 0.05). The means, standard deviations and medians were determined for the colony-forming units (Excel 2013; Microsoft, Seattle PO, USA). The numbers of surviving bacteria are presented as scatterplots. For comparison of the number of viable bacteria and those after treatment with SDF, PI and PDF + PI the nonparametric Wilcoxon test for related samples was used (*p* < 0.05).

## Results

In order to obtain preliminary information about a potential antibacterial effect of the liquids from Riva Star, a modified agar diffusion technique was used (Table 1). The largest inhibition zones were produced by undiluted silver diamine fluoride (SDF) ranging from 1.2 ± 0.1 cm (*E. faecalis* 20376) to 7.5 ± 0.9 cm (*P. micra* 20468), followed by undiluted SDF + PI ranging from 0.9 ± 0.1 cm (*E. faecalis* 20376) to 5.4 ± 0.3 cm (*P. micra* 20468). PI only produced inhibition zones in *P. micra* 20468 (0.9 ± 0.45 cm). The differences in size were statistically significant for all three bacterial strains (*p* < 0.05).

**Table 1:** Mean values and standard deviations (SD; ±) of the inhibition zones in centimeters of the three investigated strains: *E. faecalis* 20376, *A. naeslundii* A65 and *P. micra* 20468 in different concentrations of the solutions

Solution/concentration	*E. faecalis*	SD {±)	*A. naeslundii*	SD {±)	*P. micra*	SD {±)
SDF/38%	1.23	0.10	1.68	0.11	7.52	0.89
SDF/19%	0.88	0.08	1.42	0.13	5.48	0.23
SDF/9.5%	0.73	0.09	1.09	0.05	4.57	0.28
SDF/3.8%	N/A	N/A	0.95	0.15	3.48	0.31
PI/undiluted	0.0	0.0	0.0	0.0	0.90	0.45
SDF/38% + PI/undiluted	0.95	0.11	1.29	0.15	5.49	0.28
SDF/19% + PI/undiluted	0.84	0.12	1.46	0.17	4.34	0.30
SDF/9.5% + PI/undiluted	0.93	0.17	1.50	0.09	3.55	0.18
SDF/3.8% + PI/undiluted	N/A	N/A	1.50	0.10	3.25	0.18

The differences in size were statistically significant for all three bacterial strains (*p* < 0.05). Riva Star is delivered with a 38% SDF concentration

*SDF* silver diamine fluoride, *PI* potassium iodide, *SD* standard deviation, *M* median, *Max* maxima, *Min* minima; n = 6

**Fig. 2:**
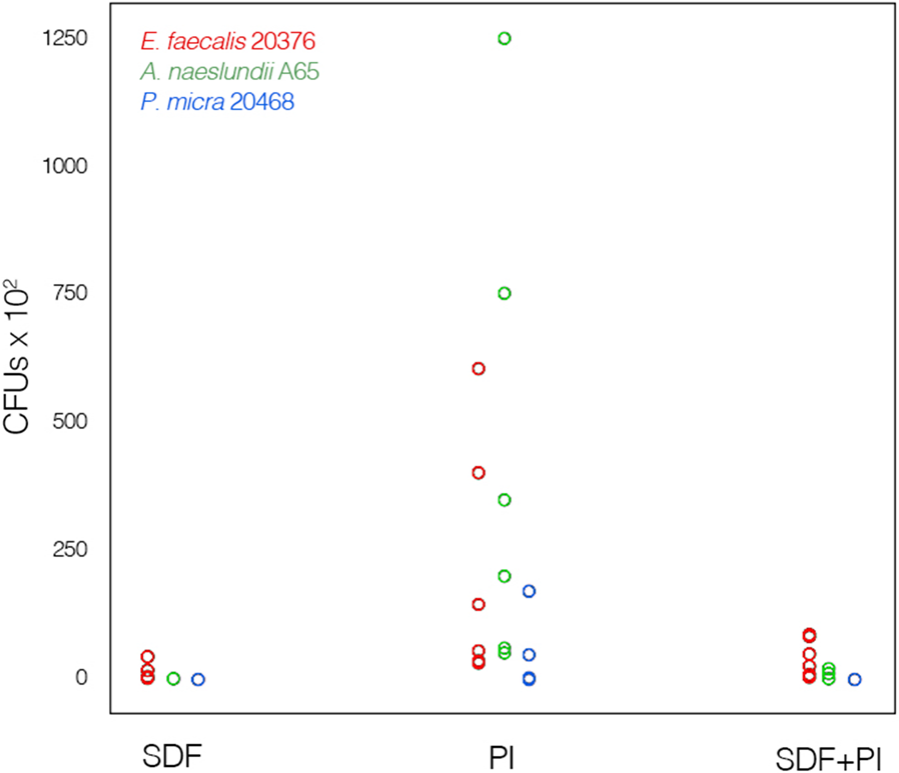
Scatter diagram of the colony-forming units with *E. faecalis* 20376, *A. naeslundii* A65 *and P. micra* 20468 after contact with SDF, PI or SDF + PI. Both in *E. faecalis* and in *A. naeslundii* the highest variation in the number of CFUs is seen after treatment with PI, and lowest for treatment with SDF. In *P. micra* after contact with SDF or SDF + PI no colonies were detected, and only a low variation in the number of CFUs can be observed after treatment with PI (*SDF* silver diamine fluoride, *PI* potassium iodide; n = 6)

After direct contact of the investigated bacterial species with the respective bactericidal solution, the numbers of viable bacteria were determined on plates containing 300 or fewer colonies as colony forming units (CFU) and are listed in Table 2 together with the numbers for the untreated controls, which are shown as scatterplot (Fig. 2). The calculated bactericidal rates in percent for each strain are also listed in Table 2.

**Table 2:** CFUs of the control (untreated culture), after being in contact with the respective solutions (SDF, PI and SF + PI) and reduction rates in percent achieved by the respective antibacterial solutions

Trial	Control	SDF	SDF (%)	PI	PI (%)	SDF + PI	SF + PI (%)
*Enterococcus faecalis* DSM 20376							
Mean	168.0 × 10^5^	10.0 × 10^2^	99.99	210.8 × 10^2^	99.86	40.9 × 10^2^	99.96
SD	98.62	16.47	0.01	238.52	0.18	36.25	0.04
M	141.5 × 10^5^	1.75 × 10^2^	99.99	98.5 × 10^2^	99.93	35.3 × 10^2^	99.98
Min	83.0 × 10^5^	0.0	99.97	29 × 10^2^	99.51	1.6 × 10^2^	99.89
Max	353.0 × 10^5^	41.5 × 10^2^	99.99	604 × 10^2^	99.98	84.8 × 10^2^	99.99
*Parvimonas micra* DSM 20468							
Mean	32.2 × 10^5^	0.0	100.00	37.9 × 10^2^	99.98	0.0	100.00
SD	22.06	0.0	0.00	68.78	0.03	0.0	0.00
M	26.4 × 10^5^	0.0	100.00	2.8 × 10^2^	99.99	0.0	100.00
Min	5.2 × 10^5^	0.0	100.00	0.0	99.93	0.0	100.00
Max	66.0 × 10^5^	0.0	100.00	173.0 × 10^2^	100.00	0.0	100.00
*Actinomyces naeslundii* A65							
Mean	150.0 × 10^5^	0.0	100.00	443.3 × 10^2^	99.73	4.8 × 10^2^	100.00
SD	67.18	0.0	0.0	472.55	0.25	8.13	0.01
M	138.0 × 10^5^	0.0	100.00	274.5 × 10^2^	99.78	0.0	100.00
Min	71.0 × 10^6^	0.0	100.00	50.0 × 10^2^	99.27	0.0	99.98
Max	269.0 × 10^6^	0.0	100.00	1250.0 × 10^2^	99.95	19.5 × 10^2^	100.00

The calculated bactericidal rate for *A. naeslundii* and *P. micra* with SDF was 100% (no CFUs could be observed in any trial). The bactericidal rate of PI as well as of SDF + PI showed relatively high bactericidal rates in this group; yet, not as effective as SDF. Riva Star is delivered with a 38% SDF concentration

*SDF* silver diamine fluoride, *PI* potassium iodide, *SD* standard deviation, *M* median, *Max* maxima, *Min* minima; n = 6

After contact of *E. faecalis* with SDF, only a relatively low number of viable bacteria could be determined; yet, in only one trial a complete bactericidal effect of SDF was observed (Table 2). For *A. naeslundii* treated with SDF, no viable cells could be detected. In four of the six trials after contact with SDF + PI no colonies could be found (Table 2). No viable cells were observed after contact of *P. micra* with SDF and SDF + PI (Table 2).

The difference in numbers of viable untreated bacteria and those after treatment with SDF, PI and PDF + PI was statistically significant for *E. faecalis*, *A. naeslundii* and *P. micra* (*p* < 0.05).

## Discussion

To the best of our knowledge, Riva Star has been investigated and clinically used for caries arrest and as a desensitizer for hypersensitive teeth. In order to be able to use SDF and/or PI as a root canal disinfecting solution, their bactericidal efficacy was investigated in the present study on bacterial species that are frequently found in infected in root canals. An in vitro methodology was developed as an approach to investigate the bactericidal effect of SDF and PI, under standardized research parameters, in an effort to avoid bias in the results obtained. The bactericidal efficacy of SDF and PI could not be assessed in this study by means of a test tube dilution test because silver, from the SDF solution, forms an insoluble precipitate with components of the liquid culture media. A suitable method for this type of research could be the agar diffusion test. This technique has been used to investigate the bactericidal effect of sodium hypochlorite and chlorhexidine against the same bacterial species (*E. faecalis*, *A. naeslundii* and *P. micra*) [25–27]. In order to test the ability of SDF to arrest the progression of carious lesions, the bactericidal effect of SDF at concentrations of 12% or 30% has been also been investigated with method, using *S. mutans* as test strain [28]; thus, it has been shown that this technique is a suitable one. However, in the agar diffusion test the contact time of the test solutions with the bacteria is at least 24 h, which is by far longer than in a clinical situation during an endodontic treatment. Therefore, a method based on the preliminary results from the agar diffusion tests, namely the sizes of the inhibition zones, was developed to investigate the bactericidal effect of the Riva Star components, simulating the clinical applicability. The possibility to apply the Riva Star components by means of an endodontic standardized syringe was not taken into consideration in this study. However, it would be advisable to consider the possibility that, in a clinical situation, the irrigating syringe opening and/or needle could clog with the solution. SDF, PI and SDF + PI were applied to bacterial colonies on paper discs with help of the provided micro brushes, and after exposure for 30 s the bacterial suspensions were serially diluted and spread on agar plates. The viable bacteria count was determined after at least 24 h of incubation (depending on the bacterial species). Such a method has, to the best of our knowledge, has not yet been described in the literature. However, Vinson et al. [29] brought also bacteria (*S. mutans*) in direct contact for 60 s with 38% SDF, PI and SDF + PI, after which a reduction in the viable count was also observed.

Different in vitro studies report about the bactericidal efficacy of commonly endodontically employed root canal irrigation solutions, such as sodium hypochlorite and chlorhexidine against *E. faecalis* [30, 31], *A. naeslundii* [26, 32] and *P. micra* [25, 31]; therefore, the bactericidal efficacy of SDF, PI and SDF + PI were also investigated using these three bacterial species. To the best of our knowledge, few reports have been published concerning the bactericidal effect of 3.8% SDF [13, 14] and PI [33] as endodontic disinfecting solutions. An effective bactericidal effect of 3.8% SDF against *E. faecalis* in root dentin [13, 14] and 3.8% SDF against *A. naeslundii* as part of a cariogenic biofilm [15, 34] has been reported. A 56% bactericidal rate of the Silver Star product SDF + PI has been reported for *S. mutans* in human dentin samples [35]. Hiraishi et al. [13], with a different research methodology, reported an effective bactericidal effect of 3.8% SDF after 15 min exposure of *E. faecalis* in biofilms formed on nitrocellulose filters. Silver nanoparticles within an alcoholic solution proved also to have a bactericidal effect against *E. faecalis*, on bovine dentin specimens after 1 week of exposure [36]. On the contrary, Spratt et al. [31] report that colloidal silver was ineffective against biofilms produced from isolates of *E. faecalis* and *P. micra* from infected root canal dentin. Although the bactericidal rates of SDF, PI and SDF + PI obtained in this investigation against *E. faecalis*, *A. naeslundii* and *P. micra* ranges from 99.27 to 100% and could also be considered as relatively high values as well as the ones reported in most studies previously mentioned [13–15, 28, 33, 34], a comparison or discussion with the aforementioned studies and ours is difficult due to the different research methodologies and parameters employed, such as exposure times of 30 s up to several days, employed. The results from the study by Vinson et al. [29], where the direct contact of the bacteria with the agents employed led to the highest reduction in viable counts by 38% SDF, followed by SDF + PI (both higher than 99.9%) and then PI (97%), resemble the ones obtain in this study, even though they studied a different bacterium (*S. mutans*).

Furthermore, no report has been published whether SDF has the ability to dissolve tissue or to remove the smear layer; thus, in our opinion for the time being, SDF and/or PI could only be recommended as enhancing disinfectant solutions (e.g. as last disinfecting procedure prior to root canal filling) and therefore, a comparison of the results obtained in this investigation with SDF, SDF + PI and PI and commonly employed root canal irrigating solutions is difficult, not only due to the different research methodologies employed, but also due to their different clinical aims. SDF showed an advantageous bactericidal effect of on *E. faecalis*, *A. naeslundii* and *P. micra*. However, a potential clinical implication of SDF in the root canal system could be a possible precipitation of silver, thus, its clinical endodontic employment should be carefully considered. It has been reported that 3.8% SDF has the ability to penetrate into the dentin tubules up to 40 µm [13] and to occlude tubular orifices even after removal of the smear layer. The authors are of the opinion that the presence of silver deposits in dentinal tubules suggest that SDF can reduce/eliminate biofilms formed in the dentinal tubules and to prevent reinfection by the relatively insoluble silver salts [13]. However, the root canal space should be thoroughly obturated with sealers and filing materials to prevent apical and coronal leakage [37]. Therefore, the employment of SDF as an irrigating solution should be carefully considered since precipitated silver could occlude areas of the complex root canal system morphology [38], such as the dentin tubules, thus, acting as a barrier to an optimum penetration of the root canal filling materials into them. This fact can be considered as a potential sealing quality hindrance for the root canal filling sealers and or thermoplastic applied gutta-percha, thus, could influence the outcome of an endodontic treatment. A further SDF endodontic employment limitation is the silver precipitation tooth discoloration potential [39, 40]. This negative side effect could be avoided through a meticulously employment of the substance. Yet, even if the bacteria in the root canal would be completely eliminated after a final irrigation with SDF, a latent discoloration potential of such tooth would conduce to a clinical aesthetic unacceptable situation, for both patient and operator. Moreover, on one hand it has been reported that SDF was cytotoxic to gingival fibroblasts in concentrations as low as 0.01% [41] and on the other hand, clinically oriented researches [42, 43] have reported mild temporary gingival reactions, such as erythema, bleeding, white changes, ulceration and pigmentation 24 h after the application of SDF. Thus, a potential accidentally caused irritating effect of SDF into the periapical tissues, especially during incomplete root formation cases, should be carefully considered when the irrigation protocol decision is taken.

## Conclusions

Silver diamine fluoride has a bactericidal effect on *E. faecalis*, *A. naeslundii* and *P. micra*.

The combination of silver diamine fluoride with potassium iodine and potassium iodine itself had a relatively lower bactericidal effect in the investigated bacterial strains in comparison with silver diamine fluoride.

## Data Availability

The datasets generated and/or analyzed during the current study are available from the corresponding author on reasonable request.

## Abbreviations

CFU: Colony forming units
DSMZ: German Collection of Microorganisms and Cell Cultures GmbH
EDTA: Ethylene diamine tetraacetic acid
PI: Potassium iodide
SDF: Silver diamine fluoride
